# Bioinspired and smart material systems for auricular cartilage engineering: toward microenvironment-responsive and self-regulating scaffolds

**DOI:** 10.1093/rb/rbag025

**Published:** 2026-03-02

**Authors:** Yan Gong, Haiyue Jiang, Xia Liu

**Affiliations:** Plastic Surgery Hospital, Chinese Academy of Medical Sciences and Peking Union Medical College, 33 Badachu Road, Shijingshan District, Beijing 100144, P. R. China; Plastic Surgery Hospital, Chinese Academy of Medical Sciences and Peking Union Medical College, 33 Badachu Road, Shijingshan District, Beijing 100144, P. R. China; Plastic Surgery Hospital, Chinese Academy of Medical Sciences and Peking Union Medical College, 33 Badachu Road, Shijingshan District, Beijing 100144, P. R. China

**Keywords:** auricular cartilage regeneration, tissue engineering, bioinspired materials, stimulus-responsive scaffolds, microenvironmental modulation

## Abstract

Auricular cartilage reconstruction remains a formidable clinical challenge due to the complex anatomical structure and limited regenerative capacity of elastic cartilage. Recent advances in tissue engineering have highlighted the potential of biomimetic and smart material systems to recreate functional auricular cartilage. This review comprehensively outlines the latest progress in the design of bioinspired scaffolds that emulate extracellular matrix composition, mechanical properties and hierarchical structures. Furthermore, it discusses emerging stimulus-responsive materials capable of sensing and adapting to environmental cues—such as temperature, pH, enzymes, light, magnetism, ultrasound and mechanical stimuli—to achieve spatiotemporally controlled regeneration. While focusing on auricular cartilage, the review also draws on concepts and strategies from broader tissue engineering domains, such as bone, skin and nerve regeneration, to inform scaffold design and functional optimization. Particular attention is given to multifunctional systems integrating self-healing, nanotechnology and intelligent release platforms, as well as strategies for modulating hypoxic, immunological and biochemical microenvironments. By bridging structural biomimicry with adaptive responsiveness and self-regulation, these advanced material systems offer promising solutions for precise, durable and patient-specific auricular cartilage reconstruction. This review aims to provide a strategic framework and future directions for the development of next-generation scaffolds tailored to the complex regenerative demands of auricular tissue.

## Graphical abstract

**Graphical Abstract:**
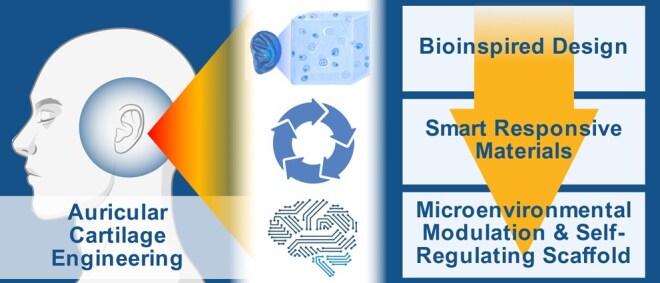

## Introduction

Auricular cartilage plays a critical role not only in sound conduction and auditory support but also in facial aesthetics and psychosocial communication [1]. Congenital microtia, traumatic injuries and tumor resections frequently lead to auricular cartilage defects, which severely impair facial symmetry, psychological well-being and quality of life, while posing substantial challenges to reconstructive surgery [2, 3]. Currently, autologous costal cartilage grafting and synthetic implants such as porous high-density polyethylene (Medpor) are the most commonly adopted clinical strategies [4]. Autologous grafts offer excellent biocompatibility and long-term integration [5], but are constrained by donor site morbidity, difficulty in shaping and risks of postoperative resorption or deformation [2]. In contrast, alloplastic implants reduce donor trauma and surgical duration, particularly in pediatric patients, but suffer from higher complication rates [5]. These limitations underscore the urgent need for safer, more customizable and functionally regenerative alternatives for auricular reconstruction [2, 6].

Tissue engineering has emerged as a promising strategy for auricular cartilage regeneration, offering the potential to recreate native-like cartilage using a combination of biomimetic scaffolds, seed cells and instructive bioactive cues [3]. Among these, the scaffold is a critical component that defines the spatial architecture, mechanical integrity and local microenvironment of the engineered tissue [7]. Recent advances in biomaterials and fabrication techniques have enabled the development of multifunctional scaffolds that integrate bioinspired design, intelligent responsiveness and dynamic microenvironmental modulation [7]. These scaffolds not only emulate the extracellular matrix (ECM) composition, mechanics and three-dimensional (3D) morphology of native auricular cartilage but also exhibit advanced functionalities such as stimulus-triggered gelation, self-healing and spatiotemporally controlled release of bioactive factors—greatly enhancing regenerative precision and long-term outcomes [7].

**Figure 1:**
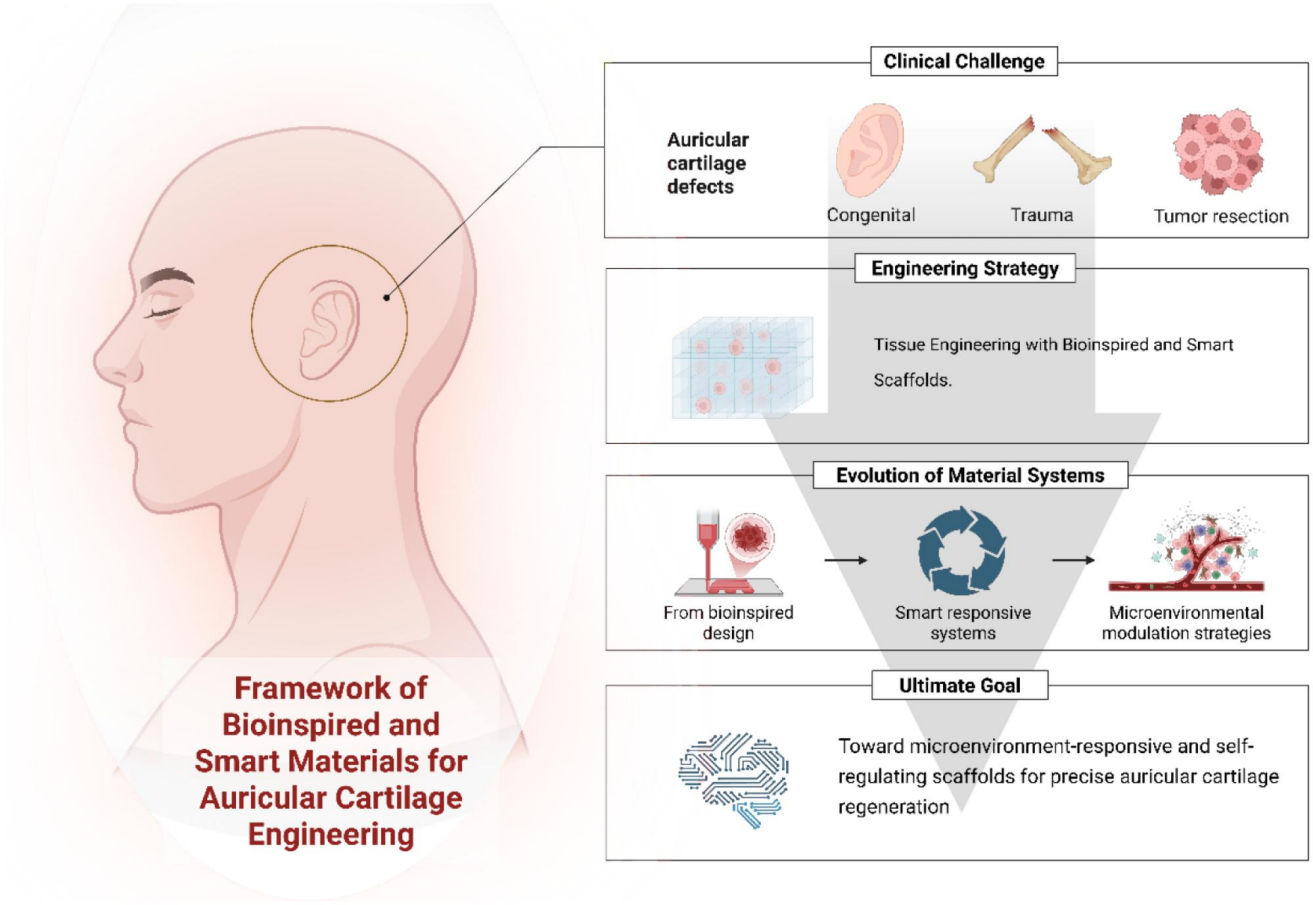
Schematic framework illustrating the evolution of scaffold strategies for auricular cartilage engineering, from structural biomimicry and smart responsiveness to microenvironmental modulation and self-regulating systems.

This review focuses on the recent progress in bioinspired and smart material systems for auricular cartilage engineering. We outline advances in ECM-mimicking materials, mechanically and structurally adaptive scaffold design, 3D/4D (four-dimensional) printing technologies and a spectrum of stimulus-responsive systems—including thermoresponsive, pH-sensitive, enzyme-degradable, light-, magnetic- and ultrasound-triggered platforms. We further highlight strategies for modulating key aspects of the regeneration microenvironment, such as hypoxia adaptation, immunomodulation and spatiotemporal delivery of growth factors. As illustrated in Figure 1, this review outlines recent developments from structural biomimicry to smart and microenvironmental modulation designs, providing a consolidated overview relevant to future scaffold development.

In this review, self-regulating scaffolds are defined as material systems that extend beyond single-trigger responsiveness and enable autonomous, feedback-driven adaptation to dynamic biological processes. Unlike conventional stimulus-responsive scaffolds that operate in an open-loop manner, self-regulating systems integrate sensing and actuation elements to achieve closed- or semi-closed-loop modulation of the regenerative microenvironment. Most current examples represent incipient or partial self-regulating platforms rather than fully autonomous constructs. A conceptual comparison between stimulus-responsive and self-regulating scaffold paradigms is provided in Figure S1.

## Biological and biomechanical basis of auricular cartilage

Auricular cartilage is a specialized elastic cartilage predominantly located in the external ear, comprising anatomical subunits such as the helix, antihelix, tragus, antitragus, scaphoid fossa and triangular fossa [8, 9]. Its complex morphology exhibits high individual variability and structural stability in 3D conformation [8]. At the histological level, auricular cartilage consists primarily of chondrocytes sparsely distributed within lacunae embedded in a dense ECM, with cellular volume representing only ∼1–5% of the total tissue [8, 10]. The ECM is predominantly composed of type II collagen, an elastin fiber network, proteoglycans (e.g. aggrecan) and glycosaminoglycans (GAGs), such as hyaluronic acid and chondroitin sulfate, providing the cartilage with unique elasticity, flexibility and compressive resilience [2, 8]. Unlike hyaline or fibrocartilage, elastic cartilage is rich in elastin, endowing the auricle with shape-memory properties and the ability to dissipate mechanical stress [2, 8, 11].

The mechanical properties of auricular cartilage are intrinsically related to its physiological function. Being a non-load-bearing cartilage, it has a relatively low elastic modulus, yet it must endure multidirectional mechanical stresses, such as compression and stretching during daily activities, while maintaining the auricle’s long-term morphological stability [2]. Human auricular cartilage exhibits site-dependent Young’s moduli typically ranging from ∼1 to 2 MPa under compressive loading [12]. Reports have indicated that depending on treatment and degradation conditions, the elastic modulus can vary between 0.8 and 8 MPa [8], highlighting its remarkable deformability and stress recovery capability [13]. This unique mechanical profile underscores the importance of matching scaffold stiffness to native auricular cartilage for successful regeneration [8]. Additionally, auricular cartilage lacks vascular, neural and lymphatic structures; thus, nutrients diffuse passively from surrounding connective tissues, resulting in low metabolic activity and limited intrinsic regenerative capacity [14]. Due to its avascularity and metabolic inactivity, injury to auricular cartilage heals slowly, often undergoing fibrosis rather than regeneration, necessitating surgical interventions for effective reconstruction [2].

Auricular cartilage regeneration involves complex, coordinated interactions among seed cells (e.g. chondrocytes or mesenchymal stem cells), ECM and microenvironmental factors [6, 7]. The ECM not only provides mechanical support but also modulates cell behavior through its biochemical composition, 3D architecture and biomechanical properties [15, 16]. The stability of the chondrocyte phenotype is highly dependent on ECM characteristics such as type II collagen abundance, fiber alignment, interconnected pore structures and mechanical stimuli including tensile or shear stresses exerted by the matrix [17]. Failure to replicate these critical ECM structural and mechanical cues in tissue-engineered scaffolds frequently leads to chondrocyte dedifferentiation, altered lineage commitment and undesirable fibrosis or ossification [18, 19].

The regulation of cellular differentiation towards chondrogenesis involves multiple signaling pathways, prominently including TGF-β/Smad, BMP/Smad, insulin-like growth factor-1 (IGF-1)/PI3K-Akt and Wnt/β-catenin cascades [20, 21]. Specifically, TGF-β1 not only induces mesenchymal stem cell (MSC) chondrogenesis but also facilitates the integration of implanted cartilage constructs into host tissue, promoting cartilage regeneration [6]. TGF-β3 is essential in early chondrogenic differentiation, activating Smad2/3 signaling to enhance SOX9 binding to Col2 enhancers, thus upregulating cartilage-specific gene expression [22]. BMP-2 primarily acts through Smad1/5/8 pathways to promote matrix production and cartilage maturation, as evidenced by impaired cartilage formation following BMP-2 deletion in embryonic models [6, 22]. IGF-1 enhances chondrocyte survival, proteoglycan synthesis and synergistically cooperates with TGF-β to augment cartilage formation [6, 23]. Aberrant activation of Wnt signaling, particularly via β-catenin, may induce hypertrophic differentiation or ossification; therefore, precise modulation of Wnt pathway activity through scaffold design or growth factor release is critical to preventing premature ossification [18, 24, 25].

Apart from ECM composition and signaling cues, auricular cartilage regeneration is significantly influenced by the physicochemical characteristics of the local microenvironment. First, auricular cartilage naturally resides in a hypoxic environment (∼1–5% O_2_) due to avascularity, where hypoxia-inducible factor-1α (HIF-1α) stabilization activates chondrogenic pathways and maintains immature chondrocyte phenotypes [26, 27]. Second, local pH fluctuations significantly impact cellular metabolism and enzyme activity; acidic environments typically inhibit chondrogenesis, while alkaline conditions might induce undesirable osteogenesis, emphasizing the need for scaffolds with inherent buffering capacity [23]. Additionally, variations in inorganic ion concentrations, such as calcium, sulfate and sodium, directly affect ECM synthesis and cell adhesion [23]. Importantly, implanted scaffolds invariably trigger local immune responses. Macrophage polarization states (pro-inflammatory M1 vs anti-inflammatory regenerative M2) profoundly influence cartilage regeneration outcomes. High ratios of M2 to M1 macrophages at early implantation stages positively correlate with improved cartilage integration and remodeling [28].

**Figure 2:**
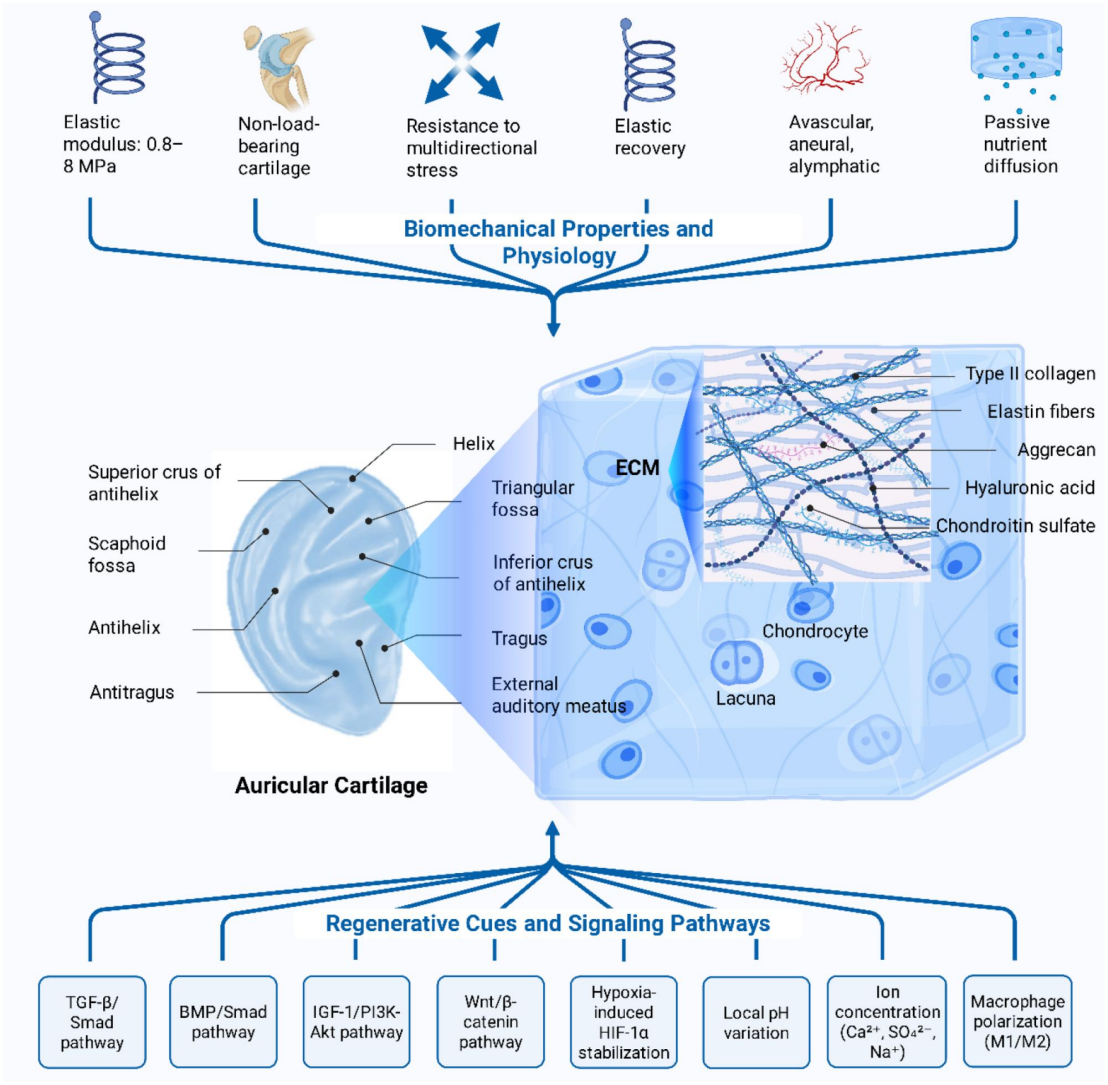
Schematic overview of the biological and biomechanical basis of auricular cartilage regeneration. Auricular cartilage exhibits a complex 3D anatomy with region-specific structural units and a unique ECM composed of type II collagen, elastin, proteoglycans and GAGs. Despite being non-load-bearing, it possesses an elastic modulus suitable for maintaining morphological stability under multidirectional stress. Regeneration is regulated by seed cells, ECM cues and key signaling pathways including TGF-β/Smad, BMP/Smad, IGF-1/PI3K-Akt and Wnt/β-catenin. Microenvironmental factors such as hypoxia (HIF-1α activation), pH, ion gradients and immune responses (macrophage polarization) critically influence chondrogenesis and integration.

Collectively, auricular cartilage regeneration represents a multifactorial process involving structural, biomechanical, biochemical and immunological factors. As summarized in Figure 2, these biological and biomechanical bases form the foundation for scaffold design strategies aimed at recapitulating native auricular cartilage and promoting functional regeneration. Thus, scaffolds that effectively emulate cartilage architecture, mechanics and microenvironmental cues—while integrating smart responsiveness and self-regulating functions—are essential for successful regeneration and represent a central research priority in current tissue engineering strategies [7]. Advances at the intersection of material science and bioengineering hold substantial promise for the development of sophisticated, microenvironmental modulation scaffolds, potentially offering safer, more effective and patient-specific solutions for auricular reconstruction in clinical practice.

Compared with conventional biomaterials or purely smart-responsive systems, bioinspired smart materials are uniquely characterized by the integration of biological design logic with adaptive, stimulus-responsive material behaviors. Rather than providing static support or isolated responsiveness, these materials are designed to interact dynamically with the regenerative microenvironment in a biologically meaningful and temporally coordinated manner. This coupling of bioinspiration and smart functionality enables self-adaptive and feedback-driven material-tissue interactions, representing a fundamental distinction from traditional scaffold-based strategies.

## From biological principles to biomimetic scaffold design

**Figure 3:**
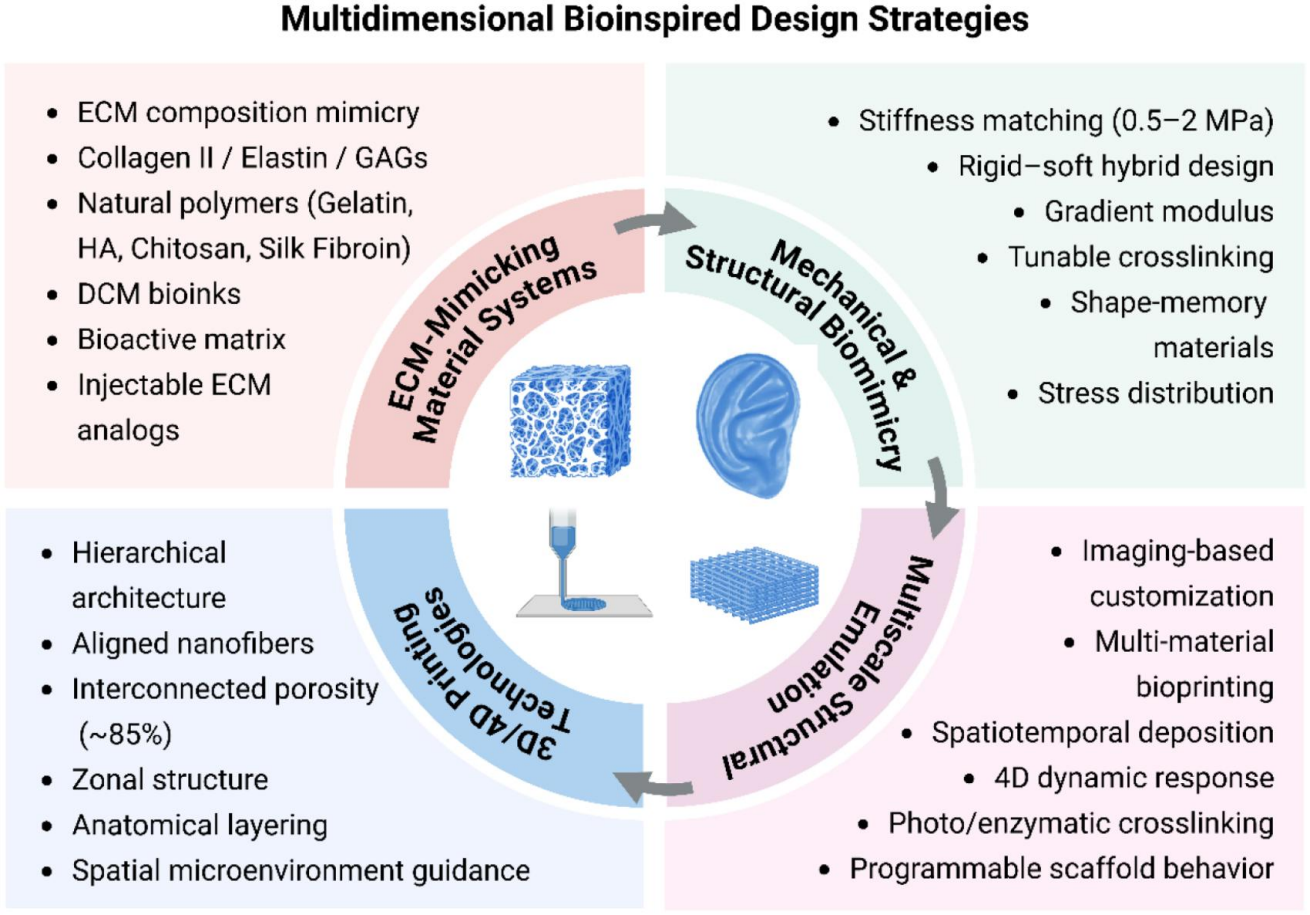
Schematic illustration of multidimensional bioinspired design strategies for auricular cartilage tissue engineering. The strategies include: (i) ECM-mimicking material systems replicating the biochemical composition of native auricular cartilage, (ii) mechanical and structural biomimicry to match the elasticity and morphological stability, (iii) microscale to macroscale architectural emulation for hierarchical organization and guided tissue remodeling and (iv) 3D/4D printing technologies for patient-specific fabrication, spatial integration and dynamic responsiveness.

Auricular cartilage is a highly specialized, avascular elastic cartilage with complex 3D morphology and refined mechanical coordination [29]. Its structural integrity and physiological function depend on the specific composition of the ECM, appropriate mechanical support and the coordinated organization of multiscale structures. Accordingly, the design of scaffolds that simultaneously replicate biochemical components, mechanical properties and tunable structures is fundamental to achieving functional auricular cartilage regeneration [30, 31]. In recent years, scaffold design in auricular cartilage tissue engineering has evolved toward multidimensional biomimicry, with a focus on four key aspects: compositional mimicry, mechanical matching, structural emulation and advanced 3D/4D fabrication. As illustrated in Figure 3, these bioinspired strategies underscore the multidimensional principles guiding current scaffold design. Importantly, unlike hyaline (articular) cartilage where load-bearing stiffness is the primary constraint, auricular cartilage engineering must prioritize an elastin-rich elastic matrix and long-term shape fidelity while actively suppressing hypertrophic maturation and endochondral ossification.

### ECM-mimicking material systems

The native auricular cartilage ECM is primarily composed of type II collagen, elastin fibers and sulfated GAGs such as hyaluronic acid and chondroitin sulfate, which collectively provide structural integrity and regulate chondrocyte function [32–34]. To replicate this highly specialized microenvironment, a variety of natural polymers have been utilized as scaffold materials, including gelatin, hyaluronic acid, chitosan, heparin [35], silk fibroin and alginate [36–38]. These biopolymers exhibit excellent biocompatibility and biodegradability and can be chemically modified, crosslinked or blended to tailor their physical properties and support chondrogenesis [39].

Hyaluronic acid-based hydrogels, due to their high-water retention and lubricating properties, are particularly favorable for elastic cartilage matrix production [37]. Silk fibroin offers superior thermal and enzymatic stability, as well as mechanical strength, making it suitable for scaffolds requiring high elasticity and shape fidelity [40]. Recent reviews have highlighted the versatility of bioengineered silk fibroin as a tissue engineering biomaterial [41]. Decellularized cartilage matrix (DCM), which retains native ECM components and spatial architecture, has emerged as a ‘tissue-derived bioink’ with remarkable capacity to induce chondrogenic differentiation and maintain phenotypic stability [42–44]. Studies incorporating DCM into 3D-printed and composite hydrogel systems have demonstrated enhanced structural fidelity and matrix deposition in auricular cartilage constructs [45, 46]. Accordingly, material selection and bioink formulation should be evaluated not only by their ability to support COL2A1/ACAN-rich cartilage formation, but also by their capacity to preserve or promote elastin assembly and to minimize fibrocartilaginous or osteogenic drift.

### Mechanical property matching

Although auricular cartilage is non-load-bearing, it must withstand multidirectional mechanical forces during facial movement while maintaining long-term structural stability. Therefore, engineered scaffolds must replicate the mechanical properties of native cartilage, particularly in terms of elastic modulus and reversible deformation [33]. While DCM-based scaffolds preserve key ECM components, their mechanical strength and shape retention are often insufficient for large-volume reconstructions. To address this, strategies such as adjusting crosslinking density, polymer content and pore architecture have enabled fine-tuning of hydrogel mechanics within a physiologically relevant range (tens to hundreds of kPa) [40, 47]. This intrinsic mechanical limitation of hydrogel-based systems, particularly GelMA, has also been widely recognized across tissue engineering applications, where excellent cytocompatibility is frequently accompanied by insufficient mechanical strength, necessitating reinforcement through composite or hybrid design strategies [48]. To facilitate direct quantitative comparison across material platforms, the representative mechanical properties of major auricular cartilage scaffold systems discussed above are summarized in Table 1. Recent work further demonstrated that reducing hydrogel stiffness in a 3D hyaluronic acid-based system enhanced the interaction of encapsulated stem cells with immobilized TGF-β3, thereby amplifying chondrogenesis via activation of the Smad and MAPK signaling pathways [51]. This finding highlights the synergistic role of mechanical and biochemical cues in optimizing chondrogenic microenvironments for auricular cartilage regeneration. Jia *et al.* proposed a composite scaffold combining cryomilled auricular cartilage matrix with gelatin, cast into a 3D-printed polycaprolactone (PCL) auricular framework and freeze-dried to produce anatomically accurate and porous constructs [33]. Similarly, polyglycolic acid (PGA)-based scaffolds demonstrated superior cartilage regeneration and mechanical performance in immunocompetent rabbit models [50]. Hydrogels based on GelMA, PVA or chitosan/gelatin/PVA blends provide both biocompatibility and support for matrix deposition [37, 38, 42]. To enhance intraoperative handling and long-term stability, hybrid strategies combining rigid polymers (e.g. PCL, PLLA, PU) with soft hydrogels have been developed, producing gradient-modulus scaffolds with superior shape retention and mechanical durability [43, 52]. For instance, Zhou *et al.* employed a multinozzle 3D printing system to fabricate auricular scaffolds combining PCL and photo-crosslinkable DCM bioinks, achieving an optimized balance between mechanical strength and biological activity [43]. Shape-memory polymers and compressible elastomers have also been introduced to enable scaffold self-recovery after deformation, preserving auricular contour postoperatively.

**Table 1:** Quantitative comparison of mechanical properties of representative auricular cartilage scaffold systems.

Scaffold system	Mechanical property (range)	Test mode and conditions	Relative to native cartilage	Key implication	Ref.
DCM-based hydrogels	Compressive modulus (**0.05–0.3 MPa**)	Compression, fully hydrated	Below range	Insufficient stiffness alone; prone to shape collapse	[33, 40, 45–47]
GelMA hydrogels	Storage modulus *G*′ (**5–50 kPa**)	Rheology, 1–10 Hz	Far below range	Highly cell-friendly but mechanically weak	[48]
GelMA composite scaffolds	Elastic/compressive modulus (**0.3–2 MPa**)	Compression or tensile, hydrated	Partially within	Tunable stiffness; balance required	[38, 42, 49]
Silk fibroin systems	Compressive modulus (**0.5–5 MPa**)	Compression, wet state	Within range	Favorable stiffness and elasticity	[40, 41]
PCL-reinforced composites	Elastic modulus (**5–20 MPa**)	Compression or bending	Often exceeds	Risk of over-stiffening and stress shielding	[33, 43, 49, 50]
3D-printed polymers	Elastic modulus (**10–100 MPa**)	Compression or tensile	Above range	Excellent shape fidelity; limited bioactivity	[45, 46, 49]

### Multiscale structural design

At the microscale, ECM-mimetic scaffolds must replicate features such as pore interconnectivity, collagen fiber alignment and hierarchical architecture to support cellular infiltration, nutrient transport and guided matrix deposition [53, 54]. For instance, Maity *et al.* used 100 μm decellularized goat auricular cartilage scaffolds with fiber spacings of 15–20 μm and 85% porosity, which doubled cartilage regeneration efficiency and achieved GAG content levels reaching 90% of native cartilage [55]. Nanofibrous scaffolds fabricated by electrospinning or microscale 3D printing techniques have been used to mimic collagen alignment and enhance directional cell migration, organization and chondrogenic differentiation [56]. Surface microtopography has also been exploited to guide cell alignment and differentiation. A representative example is a biomimetic microgrooved methacrylated silk fibroin scaffold, in which microgroove-guided stem cell organization enabled stable chondrogenic differentiation by mimicking a multilayered tissue architecture without exogenous induction [57]. Bacterial nanocellulose (BNC)/GelMA hydrogels with networked nanofibers showed enhanced GAG deposition and mechanical strength in nude mouse models, confirming the importance of fiber orientation in ECM organization [58].

At the macroscale, multilayered and zonally organized scaffolds have been developed to recapitulate the anatomical heterogeneity of auricular cartilage [59]. Integration of stratified architectures—with dense superficial, elastic middle and porous basal regions—enables simultaneous mechanical reinforcement and region-specific cellular functions, while facilitating intraoperative moldability and post-implantation remodeling [42, 56, 60]. For example, a PLA scaffold directly loaded with autologous cartilage chips and GelMA hydrogel enabled immediate intraoperative implantation, yielding over 95% morphological fidelity and elastic modulus closely matching native auricular cartilage after 3 months *in vivo* [61]. In another study, silk fibroin-PLLA microsphere composite scaffolds with longitudinal microchannels significantly enhanced cellular alignment, ECM anisotropy and spatially ordered tissue reconstruction [40]. These findings highlight the importance of hierarchical design principles that span microscale to macroscale levels for engineering anatomically and functionally robust auricular cartilage constructs.

### 3D/4D printing technologies

3D printing technologies enable precise fabrication of patient-specific auricular scaffolds based on CT/MRI data. Methods such as fused deposition modeling, digital light processing and microextrusion allow integration of natural polymers [62], synthetic materials and cell-laden bioinks into composite constructs [63–65]. Melt electro-writing offers ultrafine fiber resolution and improved mechanical stiffness (up to 13-fold), effectively reproducing patient-specific ear anatomy [66]. Multilayered and multiphase scaffolds fabricated using composite strategies (e.g. decellularized matrix particles + degradable polymers) combine mechanical tunability with biological responsiveness [43, 45]. Multinozzle printing systems enable spatial deposition of stiff and soft components alongside live cells, enhancing zonal functionality and printing resolution [42]. Composite materials such as WPU-PCL printed at low temperatures with GelMA hydrogels achieved cartilage-matching mechanical properties (0.5–1.2 MPa) and >90% cell viability [49]. Print parameters such as nozzle diameter, infill density and layer thickness directly influence scaffold microarchitecture, which in turn regulates cell adhesion, migration and ECM synthesis [43, 56, 67]. Beyond photocrosslinkable GelMA, enzymatic crosslinking (e.g. HA-TG systems using Factor XIIIa activation) has emerged as a biocompatible strategy for *in situ* gelation with enhanced stiffness and cell viability [60, 68].

Four-dimensional printing, which introduces a temporal response dimension to conventional 3D fabrication [69], enables scaffolds to undergo shape transformation or functional activation in response to environmental stimuli such as temperature, humidity, pH, light or magnetic fields [39, 67, 70]. These dynamic scaffolds are particularly suitable for constructing auricular cartilage with complex topography and functional demands. For instance, thermoresponsive hydrogels can enable delayed expansion and *in vivo* conformal fitting; magnetic or photothermal responsiveness provides remote control and sustained performance under physiological conditions [67, 70]. The intelligent evolution capacity of 4D scaffolds offers unprecedented potential to improve adaptation to host microenvironments, spatiotemporal control of regeneration and long-term functional stability—laying the foundation for structurally and functionally integrated auricular cartilage constructs.

## Enabling responsive control in biomimetic scaffolds

**Figure 4:**
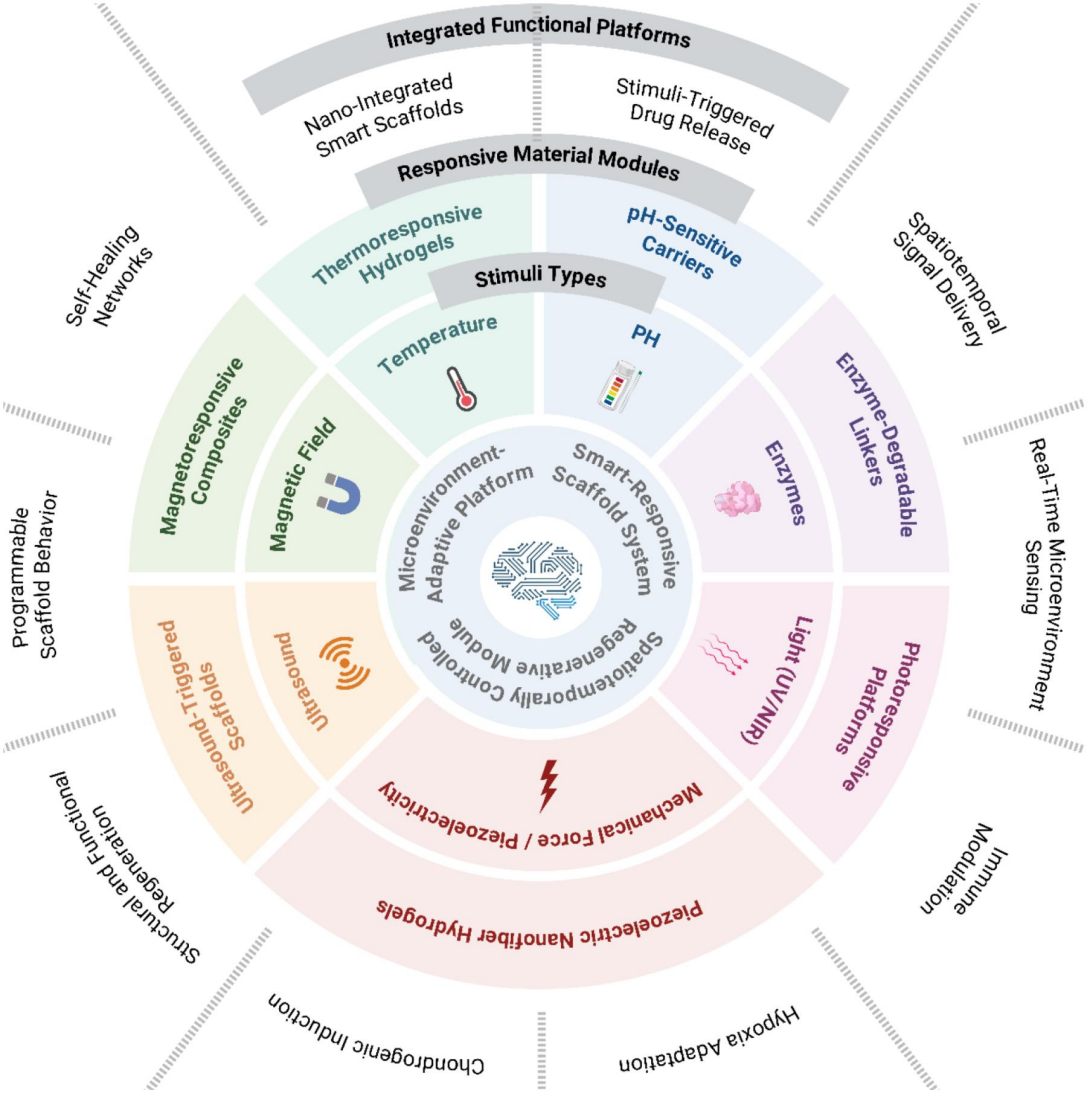
Various smart-responsive material systems offer promising strategies that can be adapted and applied in auricular cartilage tissue engineering. Environmental stimuli—including temperature, pH, enzymes, light, magnetic field, ultrasound, mechanical force and redox signals—can trigger functional responses such as phase transition, degradation, drug release or signal activation. These stimulus-responsive systems are further integrated into multifunctional composite platforms with capabilities such as self-healing, immune modulation, spatiotemporal signal delivery and structural remodeling, providing design inspiration for dynamic and microenvironmental modulation cartilage regeneration scaffolds.

The regeneration of auricular cartilage occurs within a microenvironment shaped by mechanical stimulation, inflammatory fluctuations, hypoxia and biochemical signaling [71]. Figure 4 provides an overview of the diverse stimuli and integrated functionalities underlying stimulus-responsive material systems for auricular cartilage engineering, highlighting how different external and endogenous cues are translated into spatiotemporal control of cellular behavior and matrix remodeling. To address this complexity, next-generation scaffolds must move beyond passive support to actively respond to environmental cues—enabling real-time modulation of material properties, bioactive factor release and cellular behavior. Stimulus-responsive materials, endowed with ‘sensing-responding-adapting’ capabilities, have emerged as a promising strategy to meet these demands [72]. This section highlights two major approaches: (i) stimulus-responsive systems triggered by diverse environmental signals and (ii) multifunctional composite modules integrating self-healing and nanoscale responsiveness. Together, these strategies span a broad spectrum of responsive mechanisms that can be coupled with self-healing, immune modulation and spatiotemporal signal delivery, providing a conceptual framework for designing stimulus-responsive scaffolds for auricular cartilage regeneration.

### Stimulus-responsive systems

#### Thermoresponsive hydrogels

Thermoresponsive hydrogels undergo sol-gel transitions near physiological temperatures, forming stable 3D networks *in situ*. Materials such as poly(N-isopropylacrylamide) (PNIPAm), gelatin and hydroxypropyl methylcellulose exhibit lower critical solution temperatures near 32–37°C, allowing for injectable delivery and temperature-induced gelation [39]. Systems based on PNIPAm-grafted chitosan have been co-loaded with endothelial progenitor cells, demonstrating enhanced chondrogenic induction and gelation *in situ* [20, 73]. Composite networks integrating methylcellulose oxide and chitosan oligosaccharides offer dual advantages of thermosensitivity and self-healing, while enabling stable encapsulation and release of bioactive factors [74]. These hydrogels are well suited for minimally invasive auricular reconstruction by enabling rapid *in situ* gelation and, when combined with injectable molding/printing strategies, facilitating anatomically precise ear-shaped constructs without extensive surgical sculpting.

#### pH-responsive systems

Inflammatory cartilage microenvironments often exhibit mild acidosis (pH 6.5–6.8), whereas native cartilage ECM is weakly acidic (pH ∼7.2) due to sulfated GAG-associated fixed charges [44, 75]. pH-responsive hydrogels incorporating ionizable groups (e.g. carboxyls or amines) can translate such shifts into tunable swelling, degradation or cargo release [76]. Representative pH-sensitive systems have shown inflammation-adaptive immunomodulation and stress-selective clearance of pathological cell populations in related cartilage contexts [77, 78]. These features underscore the promise of pH-responsive materials as adaptable platforms for modulating the inflammatory microenvironment and enhancing regeneration in auricular cartilage tissue engineering.

#### Enzyme-responsive hydrogels

During tissue remodeling, proteolytic enzymes such as matrix metalloproteinases (MMPs) and lysozyme are upregulated, providing bio-specific triggers for hydrogel degradation and payload release. Enzyme-sensitive systems incorporate cleavable linkages (e.g. esters, peptides, glycosides) that respond to MMPs to release therapeutic molecules such as IGF-1 or TGF-β in a temporally coordinated manner [79]. For instance, RGD-modified, MMP-9-responsive nanoplatforms promoted osteoclast apoptosis and cartilage repair in rheumatoid joints [80]. Self-assembling D-peptide nanofibers formed in response to enzymatic cleavage facilitated fibronectin remodeling and spheroid formation, demonstrating enzyme-induced dynamic microstructure reorganization [81, 82]. These features are particularly relevant to auricular cartilage engineering, where remodeling-coupled degradation can be aligned with staged matrix deposition to preserve construct shape and elasticity.

#### Photoresponsive systems

Photoresponsive materials undergo physicochemical transformations upon exposure to UV, visible or near-infrared (NIR) light. Applications include spatially defined gelation, controlled drug release and scaffold remodeling. Light-sensitive moieties—such as azobenzene, porphyrins, coumarins and nitrobenzyl derivatives—can mediate bond cleavage or conformational shifts upon irradiation [83]. NIR-responsive systems offer deep-tissue activation with minimal invasiveness. Integration of photothermal agents (e.g. gold nanorods, graphene oxide, black phosphorus) enables scaffold reshaping, localized hyperthermia and programmable drug release [84, 85]. To overcome the penetration limitations of UV/Vis light, upconversion nanoparticles (UCNPs) and downconversion nanoparticles (DCNPs) have been introduced as remote light transducers. UCNPs and DCNPs can serve as remote light transducers to enable deeper, *in situ* activation of phototriggered crosslinking or release beyond the penetration limits of UV/Vis irradiation [86]. These innovations enable deep-tissue activation of auricular scaffolds *in situ*.

#### Magnetoresponsive systems

Magnetoresponsive scaffolds embedded with superparamagnetic nanoparticles (e.g. Fe_3_O_4_, CoFe_2_O_4_) offer remote control over spatial positioning, mechanical stimulation and on-demand release of therapeutic agents. Static magnetic fields maintain scaffold alignment and shape fidelity, while alternating fields induce local hyperthermia or activate thermoresponsive and mechanoresponsive pathways. Beyond cartilage, bioinspired magnetic Janus core-shell micromotors enable magnetically guided stem cell delivery and compartmentalized vascular endothelial growth factor (VEGF) release [87]. In particular, 4D-printed Fe_3_O_4_@chitosan nanoparticle-loaded hydrogels demonstrated enhanced auricular cartilage regeneration, mechanical robustness, antibacterial activity and M2 macrophage polarization via JAK2/STAT3 signaling [70]. Complementary magnetically responsive delivery and imaging platforms further expand opportunities for controlled release and real-time monitoring in cartilage tissue engineering [88–90].

#### Ultrasound-responsive systems

Low-intensity focused ultrasound (LIFU) enables noninvasive and deep-penetrating control over smart scaffolds via mechanisms such as microbubble oscillation, localized hyperthermia and acoustic streaming [91–93]. Notably, piezoelectric nanofiber-reinforced hydrogels doped with amino-modified barium titanate have demonstrated ultrasound-triggered electrical stimulation and drug release, enhancing MSC differentiation and promoting auricular cartilage regeneration [94]. Related ultrasound-triggered platforms developed in other tissue contexts further support the versatility of acoustic activation mechanisms, providing transferable design principles for auricular constructs [95–97]. These studies highlight the versatility of ultrasound-responsive materials in diverse tissue contexts and underscore their translational promise for spatiotemporally controlled auricular cartilage engineering.

#### Piezoelectric systems

Piezoelectric materials convert mechanical deformation into local electric potentials, enabling ‘self-powered’ modulation of cellular behavior. Injectable composite hydrogels containing PLLA nanofibers and collagen have been shown to induce local voltage gradients upon ultrasound stimulation, triggering TGF-β1 release and chondrogenic differentiation in cartilage defect models [98]. In auricular reconstruction, such platforms could simultaneously modulate cell migration, matrix production and drug release, forming an integrated mechanoelectrical-biochemical control system.

#### Cross-system comparison of stimulus-responsive platforms

From a comparative standpoint, different stimulus-responsive systems exhibit distinct advantages and limitations in terms of activation efficiency, safety and tissue penetration depth. Thermoresponsive and enzyme-responsive systems rely on endogenous physiological cues, offering superior biocompatibility and safety, but often suffer from limited spatial precision and slower response kinetics. pH-responsive platforms enable inflammation-adaptive regulation; however, subtle pH variations in cartilage tissues may constrain their responsiveness. In contrast, exogenous-stimulus-driven systems, including photo-, magnetic- and ultrasound-responsive platforms—provide higher activation efficiency and spatiotemporal controllability. Among these, NIR light and ultrasound demonstrate greater penetration depth than UV or visible light, making them more suitable for thick or encapsulated cartilage constructs. Magnetic systems offer excellent remote controllability and repeatability but require the incorporation of magnetic nanoparticles, raising long-term biosafety considerations. Overall, no single stimulus-responsive strategy is universally optimal for auricular cartilage regeneration. Instead, hybrid systems that integrate endogenous responsiveness with externally triggered modulation may offer a balanced solution, achieving both safety and precise spatiotemporal control. Collectively, while stimulus-responsive strategies provide powerful spatiotemporal control, their clinical applicability largely depends on whether the triggering cues can be safely and reliably coupled to physiological conditions rather than external intervention.

### Multifunctional composite modules: integrated platforms for structural support, self-healing and stimulus-responsive control

Beyond listing individual self-healing or nano-enabled components, integrated functional platforms emphasize their system-level coupling—i.e. how structural robustness, delivery kinetics and microenvironmental feedback are coordinated within a single scaffold architecture. Auricular cartilage regeneration requires scaffold materials that not only replicate the physical architecture of native tissue but also dynamically adapt to complex biochemical cues and mechanical challenges. Building upon single-function stimulus-responsive systems, multifunctional composite modules represent an advanced strategy that enables synchronous control of mechanical integrity, biological signaling and microenvironmental adaptation. These platforms typically integrate self-healing mechanisms, nanoscale responsiveness, spatiotemporal signal delivery and hierarchical structural design to function as intelligent and adaptive scaffolds.

#### Self-healing systems

Microdamage from physiological loads or post-implantation remodeling—such as fiber fracture, interfacial delamination or network collapse—can progressively compromise scaffold performance [82]. Self-healing hydrogels mitigate this through autonomous repair enabled by reversible bonding networks [99]. Self-healing behavior in biomaterials can arise from diverse design paradigms, ranging from dynamic network-level interactions to molecularly engineered polymer architectures enabling reversible, stimulus-dependent adaptive responses. Dynamic covalent chemistries (e.g. imine/Schiff base, disulfide bonds, Diels–Alder adducts) allow repeatable network reformation [100], while noncovalent interactions (e.g. hydrogen bonding, ionic interactions, π–π stacking, host–guest inclusion) offer rapid and reversible healing under mild conditions [101, 102]. Beyond hydrogel-based systems, chemically programmed block and triblock copolymers, such as l-histidine–derived poly(histidine methacrylamide) architectures, have been reported to exhibit reversible pH- and temperature-responsive behavior via ionization-state switching, representing a molecular-level route toward smart, adaptive and self-regulating polymer systems [103, 104]. Collectively, these paradigms broaden the design space for self-healing and adaptive regenerative scaffolds. Advanced designs incorporate dual dynamic networks—combining covalent and physical bonds—to balance repair efficiency with mechanical robustness. These materials not only restore physical properties after damage but also preserve bioactive functions (e.g. growth factor release, cell adhesion) critical for tissue integration over long periods [105]. For instance, composite hydrogels formed by polyacrylamide and poly(styrene–acrylic acid) nanoparticles exhibit exceptional mechanical resilience and can dynamically reorganize their structure via hydrogen bonding—even after being fragmented into pieces [106]. Other strategies for constructing reconstructable scaffolds include hybrid systems based on carboxymethyl chitosan, oxidized alginate and polyacrylamide [107], as well as host–guest networks using β-cyclodextrin and deoxycholic acid conjugates to achieve reversible crosslinking and dynamic remodeling [108]. These self-healing systems offer promising solutions for maintaining scaffold functionality under physiological stress and ensuring long-term tissue integration in auricular cartilage regeneration. From a design standpoint, network-based self-healing hydrogels are generally more suitable for maintaining structural continuity under repetitive deformation, whereas molecularly engineered polymer systems offer greater programmability but may face higher translational barriers due to synthetic complexity.

#### Nano-integrated smart systems

To meet the dynamic and multifactorial demands of auricular cartilage regeneration, nano-integrated smart systems have emerged as advanced scaffold platforms that endow materials with environmental sensing, spatiotemporal precision and functional adaptability [109]. By embedding functional nanomaterials—such as superparamagnetic iron oxide nanoparticles (SPIONs), UCNPs, carbon dots (CDs) or metal-organic frameworks (MOFs)—into hydrogel or polymer matrices, these scaffolds respond to physical (e.g. magnetic field, NIR light), chemical (e.g. pH, ROS) or biological (e.g. MMPs, lysozyme) stimuli, enabling targeted release of bioactive agents and real-time modulation of the local microenvironment. For instance, SPION-loaded hydrogels allow magnetically guided shape retention and remote activation, while also promoting M2 macrophage polarization via JAK2/STAT3 signaling, supporting inflammation resolution and chondrogenesis in auricular reconstruction [70]. UCNPs facilitate noninvasive, deep-tissue phototriggered drug release, overcoming light penetration limitations and enabling precise *in vivo* control [110]. MOFs serve as pH- and enzyme-sensitive carriers with high drug-loading capacity and catalytic functionality [101, 111, 112], while CDs enable redox-sensitive release and multichannel fluorescent tracking, supporting integrated therapeutic and diagnostic (theranostic) applications [113].

These nano-composite platforms also contribute to mechanical reinforcement and cellular regulation by modulating scaffold stiffness, topography and adhesion dynamics [109]. Strategies such as combining magnetic actuation with piezoelectric signaling or light-heat conversion with drug release have yielded synergistic effects in promoting stem cell recruitment, chondrogenic differentiation and anti-inflammatory responses [70, 114]. Moreover, smart nanosystems incorporating carbon-based dots, ROS-sensitive carriers or enzyme-degradable linkers have demonstrated potential in temporally controlled release of chondrogenic factors such as TGF-β, IGF-1 or collagen peptides [115]. Looking forward, the convergence of multifunctional nanoparticles with responsive hydrogels, imaging probes and bioactive delivery systems offers unprecedented potential to construct ‘sense-respond-adapt’ scaffolds tailored to the anatomical complexity and immunometabolic environment of auricular cartilage. As a result, nano-integrated smart materials are not only structural components but also active regulators—forming a new generation of programmable, adaptive and theranostic scaffolds for precise auricular cartilage regeneration.

#### Integrated functional platforms: structural support, signal delivery and microenvironmental modulation

Next-generation auricular cartilage scaffolds are being engineered as integrated platforms that synchronize mechanical support, bioactive signaling and microenvironmental modulation. Unlike passive frameworks, these scaffolds act as bio-instructive systems, capable of actively coordinating structural integrity with immune and regenerative processes. To replicate the native auricle, scaffolds must match its elastic anisotropy and complex morphology while providing long-term mechanical stability. Concurrently, embedded delivery systems—ranging from microspheres to stimulus-responsive linkers—enable the spatiotemporally controlled release of chondrogenic and immunomodulatory cues, such as TGF-β3, BMP-2 and IL-4, to direct cell differentiation and matrix deposition [116–119].

At the microenvironmental level, scaffolds incorporating hypoxia-adaptive components, pH-buffering agents or ROS-scavenging nanomaterials can dynamically adjust local biochemical conditions to suppress fibrosis and ectopic ossification while supporting cartilage-specific lineage commitment [120, 121]. Furthermore, emerging designs integrate multimodal feedback mechanisms that respond to changes in inflammation, oxygenation or mechanical stress—enabling real-time modulation of scaffold behavior [122, 123]. This convergence of structural mimicry, intelligent signal delivery and adaptive regulation represents a pivotal advance toward self-regulating, clinically translatable auricular cartilage scaffolds.

## From stimulus-responsive scaffolds to microenvironmental regulation of regeneration

**Figure 5:**
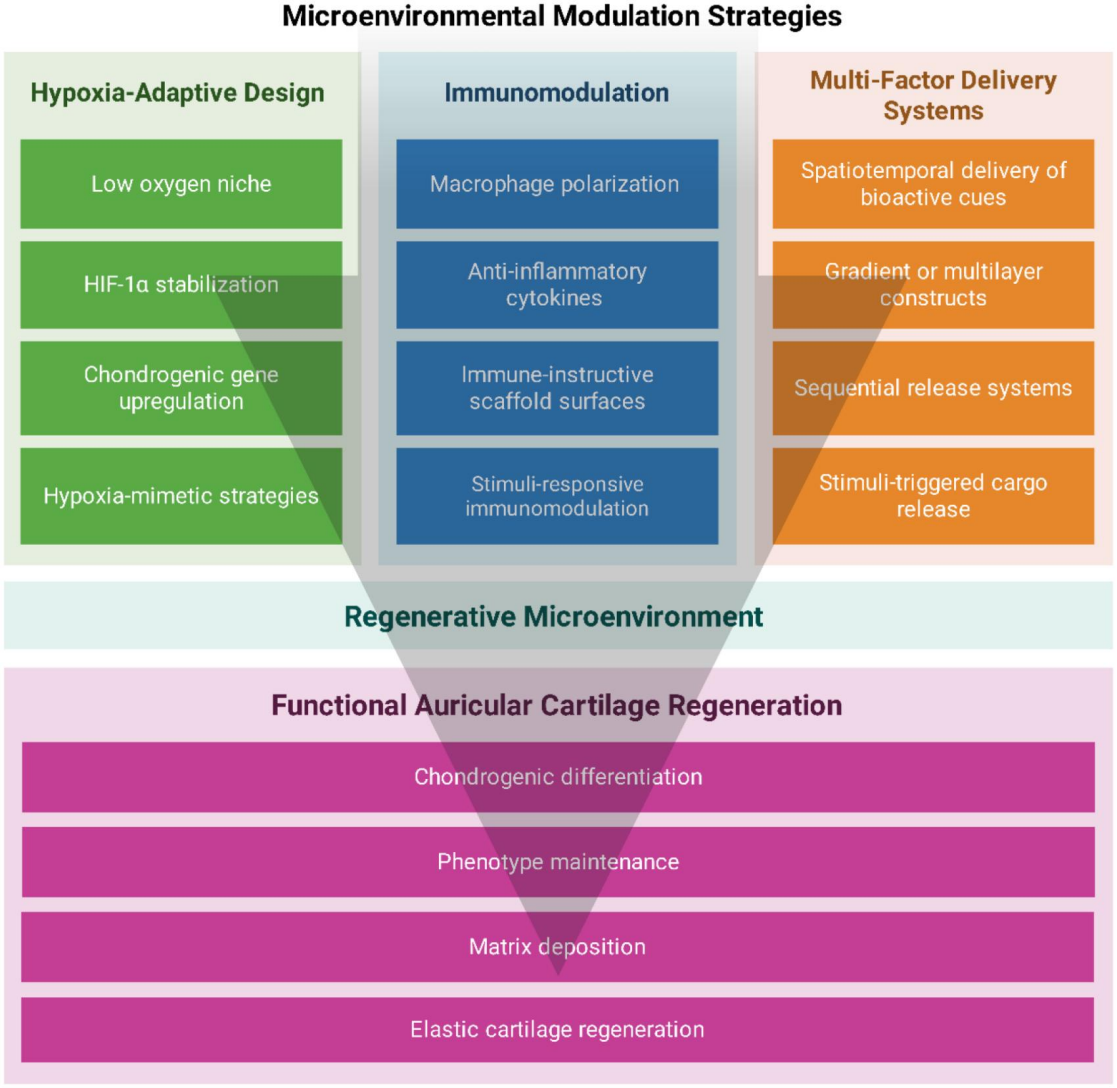
Schematic illustration of three key microenvironmental modulation strategies for auricular cartilage reconstruction: (i) hypoxia-adaptive designs that stabilize HIF-1α and promote chondrogenic gene expression, (ii) immunomodulatory platforms that guide macrophage polarization and inflammatory resolution and (iii) spatiotemporal multifactor delivery systems for staged release of bioactive cues. Together, these strategies establish a regenerative microenvironment that supports phenotype maintenance, matrix deposition and functional auricular cartilage formation.

Successful auricular cartilage regeneration depends on microenvironmental regulation in addition to biomimetic scaffold design and bioactive signaling [124]. For elastic cartilage, phenotype maintenance requires sustaining an elastin-rich ECM while preventing hypertrophy-associated ossification driven by vascular invasion, inflammatory cues or excessive osteoinductive signaling. As illustrated in Figure 5, three representative strategies are discussed in this section: hypoxia-adaptive design, immunomodulatory platforms and spatiotemporally controlled multifactor delivery [125].

### Hypoxia-adaptive design

Cartilage is physiologically avascular and hypoxia-tolerant, and auricular cartilage typically resides in a 1–5% oxygen niche [2, 26]. Maintaining hypoxia supports chondrocyte phenotype and anabolic activity while suppressing fibroblast invasion and osteogenic transdifferentiation, thereby preserving the architecture and function of regenerating auricular cartilage [26].

#### Biological roles of hypoxia in cartilage regeneration

Under hypoxia, HIF-1α becomes stabilized and acts as a master transcriptional regulator of chondrogenesis [126]. HIF-1α upregulates key cartilage-related genes such as SOX9, COL2A1 and ACAN, enhances glycolytic metabolism to sustain energy under low oxygen and protects cells from oxidative stress and apoptosis—thereby improving the survival and functional persistence of chondrocytes and MSCs [27]. Moreover, hypoxia attenuates angiogenesis and the expression of osteogenic inducers such as VEGF and RUNX2, helping to maintain the avascular phenotype of auricular cartilage and reducing the risk of ectopic ossification [127]. This anti-angiogenic, anti-ossification effect is particularly relevant for auricular reconstruction, where any shift toward hypertrophy compromises elasticity and shape retention, whereas vascularization is often beneficial or unavoidable in other regenerative contexts. Aberrant angiogenesis leads to excessive oxygen exposure, which destabilizes HIF-1α in chondrocytes and accelerates cartilage degeneration [128]. Beyond externally imposed hypoxia, cell-adaptive hydrogels can autonomously establish hypoxic niches that couple metabolic reprogramming (e.g. glycolysis-linked histone lactylation) with transcriptional control, with miR-455 family members further reinforcing chondrogenic programs via HIF signaling modulation [129, 130]. Accordingly, engineering scaffolds with the capacity to dynamically regulate oxygen tension represents a key strategy in auricular cartilage tissue engineering.

#### Material-based strategies for hypoxia-adaptive microenvironments

Recent strategies for engineering hypoxia-adaptive microenvironments in cartilage tissue engineering can be broadly categorized into three types: physical barrier architectures, chemically induced hypoxia simulation and bidirectional oxygen regulation systems, among which the latter begin to approach self-regulating behavior through feedback-mediated oxygen modulation.

Physical approaches utilize dense or multilayered hydrogel architectures to restrict oxygen diffusion, thereby forming localized hypoxic niches. For example, core-shell structures with a compact outer layer encapsulating a porous interior have demonstrated the ability to isolate embedded cells from ambient oxygen and preserve chondrogenic phenotypes under hypoxic stress [131]. Decreasing pore size or porosity enhances oxygen restriction but may hinder nutrient transport; thus, structural designs often integrate degradable microchannels or angiogenesis-permissive zones to reconcile oxygen regulation with metabolic support [132, 133].

Chemical strategies employ hypoxia-mimetic agents such as cobalt chloride, deferoxamine and dimethyloxalylglycine to stabilize HIF-1α and activate downstream hypoxia-responsive genes. These agents enable temporally controllable hypoxic cues, facilitating early-stage cell survival, stem cell commitment and VEGF-mediated vascularization [133].

Beyond standalone hypoxia induction, increasing attention has been directed toward the synergistic integration of HIF stabilizers with smart material platforms. When embedded within stimulus-responsive hydrogels or nanocarrier systems, HIF stabilizers such as deferoxamine or DMOG can be released in a temporally programmed or feedback-coupled manner, enabling sustained HIF activation without continuous chemical exposure. This integration allows hypoxia signaling to be coordinated with other microenvironmental cues, including immunomodulation, mechanical adaptation or growth factor delivery. Such synergistic designs transform HIF stabilization from a static hypoxia-mimetic intervention into a dynamic regulatory module within self-adaptive cartilage engineering systems, offering improved safety and long-term phenotypic stability [134, 135].

Bidirectional oxygen regulation platforms have been developed to address the dynamic oxygen demands across different stages of tissue regeneration. By incorporating oxygen-generating components (e.g. CaO_2_, MgO_2_, perfluorocarbons) alongside oxygen-consuming enzymes such as glucose oxidase or catalase, these systems enable feedback-mediated regulation of local oxygen tension. As cellular metabolism increases and oxygen levels decline, oxygen-generating reactions are activated to support cell viability; conversely, restoration of oxygen tension suppresses further oxygen generation, allowing the system to autonomously transition toward a hypoxic state that favors chondrogenic signaling. This negative feedback behavior distinguishes such platforms from single-trigger hypoxia-responsive systems [136, 137]. Additionally, spatially graded or biphasic scaffolds mimic the physiological oxygen gradient of native cartilage development—from an oxygen-rich surface to a hypoxic core—thereby promoting phenotypic stability and matrix deposition during tissue maturation [131, 136].

In this context, most physical and chemical hypoxia-inducing strategies function as stimulus-responsive, open-loop systems, whereas bidirectional oxygen-regulating scaffolds represent an incipient form of self-regulating design that adapts oxygen availability based on downstream biological responses. Despite this progress, challenges remain in achieving precise spatiotemporal oxygen control, integrating hypoxia regulation with other smart modules (e.g. immunomodulation and drug delivery) and validating long-term effects in large-animal models.

### Immunomodulation in auricular cartilage engineering

Immunomodulation critically shapes scaffold integration and long-term matrix remodeling in auricular cartilage engineering, despite the tissue’s relatively avascular and immune-privileged characteristics [4, 28, 121]. Upon scaffold implantation, innate immune cells infiltrate the site, triggering an acute inflammatory cascade that can either support or hinder regeneration depending on its timing and resolution [4]. In most reported platforms, immunomodulation is achieved through stimulus-responsive mechanisms; however, systems that couple inflammation sensing with feedback-mediated immune regulation begin to approach self-regulating behavior.

Macrophages are central mediators in this process, exhibiting remarkable plasticity between pro-inflammatory (M1) and pro-regenerative (M2) phenotypes [138–140]. M1 macrophages secrete TNF-α and IL-1β, contributing to tissue damage and matrix degradation, whereas M2 macrophages release IL-10 and TGF-β, facilitating ECM deposition and tissue remodeling [115]. Intriguingly, transient M1 activation has been shown to stimulate early chondrogenic events—such as type II collagen and aggrecan synthesis—followed by a spontaneous shift to M2 dominance around day 7, reflecting a critical inflammation-to-regeneration transition phase [115]. Thus, immunoinstructive scaffold design must aim to promote M2 polarization while avoiding prolonged M1-mediated inflammation [77].

Even bioengineered constructs fabricated with autologous chondrocytes (e.g. HATG bioinks) are not immune to host immune activation. In immunocompetent models, such constructs can elicit local inflammation, leading to fibrosis and loss of cartilage-specific ECM components [141]. Environmental context also plays a pivotal role: auricular niches support cartilage maturation and phenotype retention, while ectopic environments such as subcutaneous sites often provoke inflammatory responses and dedifferentiation of implanted chondrocytes [142]. These findings underscore that scaffold systems must be designed not only for passive biocompatibility but for active immune modulation—transforming the host response from an obstacle into a regenerative ally.

In this context, emerging immunomodulatory strategies—including biofunctional surface modification, responsive scaffold platforms, nanodelivery systems and synergistic physical cues—are enabling increasingly precise control over local immune states. By orchestrating spatiotemporal macrophage phenotypic transitions, suppressing excessive inflammation and enhancing immune-tissue integration, these approaches are laying the foundation for long-term functional regeneration of engineered auricular cartilage.

#### Surface modification and biofunctionalization

Surface-engineered scaffolds can directly influence macrophage behavior. IL-4 delivery has been shown to enhance cartilage matrix formation and chondrocyte phenotype retention in subcutaneous environments [47]. Functional coatings incorporating anti-inflammatory agents such as quercetin [143], rutin, TGF-β1 or IL-10 can establish early-stage immunosuppressive niches via covalent immobilization or layer-by-layer assembly [80]. Proteoglycan-4 was recently reported to facilitate M2 polarization and suppress inflammation, accelerating wound closure and promoting chondroprogenitor activation [144]. Natural polymers such as gelatin, chitosan and hyaluronic acid exhibit intrinsic immunoregulatory properties, modulating M2-associated gene expression (e.g. CD206, Arg-1) to support regenerative polarization. Additionally, microstructural design—such as fiber alignment, surface grooves and pore gradients—can guide immune cell chemotaxis and adhesion, presenting new avenues for scaffold-immunity crosstalk optimization [145].

#### Intelligent immunomodulatory scaffolds

Smart biomaterials with dynamic response capabilities enable microenvironmental modulation immunomodulation. For instance, PVA-gelatin scaffolds reinforced with silica nanoparticles activate the PI3K/Akt pathway to enhance chondrocyte proliferation while directing M2 macrophage polarization via multiscale porosity design [146]. Responsive immunomodulatory scaffolds incorporating enzyme-cleavable peptides (e.g. MMP-sensitive) or ROS-/pH-labile linkers enable inflammation-triggered release of anti-inflammatory cues [142]. Importantly, in systems where the released immunomodulators suppress pro-inflammatory signaling and reduce the expression of inflammatory enzymes or ROS levels, the original trigger is attenuated, establishing a negative-feedback loop. Such inflammation-sensing–immunomodulating platforms therefore move beyond single-trigger responsiveness and exhibit incipient self-regulating behavior by autonomously adjusting immune activity in response to downstream biological outcomes. Advanced platforms employing dual-responsive elements (e.g. ROS/pH-sensitive) or embedded variable structures have achieved spatial-temporal regulation of immune states [142, 147].

#### Nanodelivery systems for targeted immunointervention

Nanocarriers such as PLGA, liposomes, polymeric micelles or MOFs enable precise delivery of anti-inflammatory drugs, siRNAs, miRNAs or gene-editing tools to specific immune cells (e.g. macrophages, dendritic cells) [82, 120, 148]. Stimulus-responsive nanoplatforms further allow cargo release to be modulated by local inflammation status, offering spatiotemporally refined immunoregulatory interventions [147, 149]. One study designed an inflammation-sequential nanodelivery system to temporally release immunosuppressive peptides and chondrogenic cues in osteoarthritic lesions, significantly enhancing cartilage repair [147]—highlighting the potential of ‘immune-regeneration coupled’ systems in auricular cartilage engineering.

#### Synergistic external stimuli

Physical stimuli can be integrated into scaffold platforms to further regulate immune responses. Sustained release of low-dose glucocorticoids can attenuate acute inflammation post-implantation [150]. LIFU increases cell membrane permeability, facilitating anti-inflammatory drug uptake [151, 152]. Magnetic stimulation guides cell alignment and triggers protective signaling via mild hyperthermia, while NIR light can activate photothermal agents to suppress pro-inflammatory factors and upregulate heat shock proteins [88–90]. Future scaffold designs may integrate these stimuli into closed-loop systems that achieve ‘stimulus recognition–precision release–adaptive regulation’ for personalized immune control.

From biofunctional surfaces and stimulus-responsive scaffolds to targeted nanodelivery and synergistic stimulation, a growing arsenal of strategies is enabling refined control over host immune dynamics. In this context, most current approaches function as stimulus-responsive, open-loop systems, whereas inflammation-sensing platforms that actively suppress inflammatory triggers through immune feedback represent an incipient form of self-regulating scaffold design.

### Spatiotemporally controlled multifactor delivery systems

Auricular cartilage regeneration is a temporally and spatially coordinated process encompassing cell recruitment, lineage commitment, ECM deposition and tissue remodeling, all of which are governed by tightly regulated bioactive cues, including growth factors, transcriptional regulators, small molecules and noncoding RNAs [152–157]. Consequently, single-factor interventions are often insufficient to recapitulate native developmental programs, motivating the use of spatiotemporally controlled multifactor delivery systems to guide staged chondrogenesis and construct maturation. Commonly employed cues include the TGF-β superfamily (e.g. TGF-β1, TGF-β3), BMPs (BMP-2, BMP-7, BMP-9, BMP-5), IGF-1, VEGF, SOX9, as well as emerging regulators such as lactoferrin (LF), cadherin-11 (CDH11), desmin and retinoic acid (RA) signaling pathways. Importantly, in the context of auricular elastic cartilage, these signals must be interpreted through a phenotype-preserving lens: promoting chondrogenesis alone is insufficient unless hypertrophic maturation and ossification (e.g. RUNX2/COL10A1/MMP13-associated pathways) are simultaneously restrained.

Across regeneration stages, bioactive cues can be functionally grouped according to their dominant roles. During early lineage commitment, TGF-β3 serves as a master inducer of chondrogenesis by activating SOX9 and downstream ECM genes such as COL2A1 and ACAN and is therefore widely used for early priming of implanted progenitor cells [115, 117, 152, 158, 159]. SOX9 further functions as a central transcriptional node that promotes cartilage ECM synthesis while repressing osteogenic and catabolic programs, and its activity can be reinforced through gene delivery or SOX9-enhancing factors such as LF [156, 160–164]. During matrix production and maturation, BMP family members complement TGF-β signaling: BMP-2 enhances ECM deposition while mitigating hypertrophic drift [165, 166], BMP-9 exhibits strong chondrogenic potency in auricular progenitors [153] and BMP-5 appears to preferentially regulate elastic cartilage proliferation and regional growth [154]. In mid-to-late stages, IGF-1 supports chondrocyte survival, matrix synthesis and mechanical strengthening, and is often delivered sequentially following TGF-β3 priming [117, 155, 167–169]. Beyond canonical growth factors, elastic-cartilage-specific stabilization is further supported by LF, CDH11, desmin and RA signaling, which collectively enhance elastin-rich matrix organization, mechanical durability and auricular shape fidelity [21, 156, 157, 170, 171].

To implement these staged signals, delivery architectures such as PLGA microspheres, chitosan nanoparticles and multilayer or gradient scaffolds enable sequential or region-specific factor presentation while preserving bioactivity [117, 172, 173]. In this context, macrophage-inspired magnetic silk fibroin porous microcarriers have been developed for exosome enrichment and delivery, in which the porous architecture supports efficient loading and sustained release, while co-delivered bioactive ions contribute to pro-regenerative microenvironmental modulation [174]. Stimulus-responsive materials sensitive to enzymes, pH or temperature further allow on-demand release tailored to local microenvironments; for example, differential crosslinking in dual-layer hydrogels has enabled staged TGF-β3/IGF-1 delivery with improved ECM deposition and structural integrity [117]. In parallel, programmable non-protein cues—including small molecules (e.g. kartogenin, SP600125) and transcription factor delivery (e.g. Runx1 via PAMAM nanocarriers)—offer increased stability and additional regulatory leverage for phenotype control [175–181]. Despite these advances, challenges such as unpredictable factor interactions, limited *in vivo* monitoring of release kinetics and protein instability continue to hinder clinical translation. Future efforts should therefore prioritize integration with stimulus-responsive systems for closed-loop control, coupling with hypoxia and immune modulation, development of *in situ* sensing tools and alignment with scalable formats such as bioinks and prefabricated hydrogels. Collectively, spatiotemporally controlled multifactor delivery systems represent a critical step toward evolving auricular cartilage scaffolds from passive supports into intelligent, developmentally informed therapeutic platforms.

## Translational perspectives and *in vivo* challenges

Despite rapid advances in bioinspired and smart material systems for auricular cartilage engineering, their translational readiness remains limited. Most stimulus-responsive hydrogels, nano-integrated platforms and self-healing systems have been validated primarily in small-animal models, which provide mechanistic insight but fail to recapitulate the biomechanical scale and long-term immune–material interactions relevant to human auricular reconstruction [182]. Only a small subset of ECM-derived hydrogels and composite scaffolds have progressed to immunocompetent rabbit auricular defect models, while validation in clinically relevant large-animal models such as porcine or ovine remains scarce, constituting a key translational gap.

Beyond anatomical scaling, the long-term stability and biosafety of smart material functions remain insufficiently characterized. Repeated self-healing behavior, durability of magneto- or photo-responsive features and the *in vivo* fate of incorporated nanomaterials are rarely assessed beyond short implantation periods, raising concerns regarding immune activation and material accumulation [183]. As highlighted in recent analyses of fibrous biomaterials and multifunctional hydrogel systems, successful translation will likely require a balance between functional sophistication and material simplicity, rather than maximal integration of responsive elements [184, 185].

While multifunctional smart scaffolds enable integrated responsiveness and therapeutic activity, increasing functional integration inevitably introduces manufacturing and regulatory constraints that directly impact translational feasibility. Each additional functional layer adds critical quality attributes that must be controlled under GMP conditions, with nano-enabled systems being particularly sensitive to particle size, surface chemistry and dispersion stability—factors that can substantially affect mechanical performance, release behavior and batch-to-batch consistency. The inclusion of proteins or other biologics further restricts sterilization options and often necessitates aseptic processing, increasing process complexity and cost [186]. From a regulatory perspective, highly integrated platforms combining multiple triggers and cargos may obscure the principal mode of action and complicate risk assessment, especially when long-lived inorganic components are involved. Designs in which individual functions correspond to clearly defined and independently verifiable endpoints—such as mechanical integrity, release kinetics or responsiveness thresholds—are therefore more amenable to qualification than tightly coupled, emergent behaviors. Consequently, scaffold systems with fewer coupled variables and standardized control points, including ECM-derived or clinically familiar polymer matrices and nanocomposites used primarily for mechanical reinforcement or single-function actuation, are generally more suitable for near-term translation. In contrast, platforms requiring repeated external stimulation or multibiologic, staged release, as well as fully integrated sensing–feedback systems, face substantially higher barriers due to challenges in standardization, validation and manufacturing reproducibility.

## Conclusion and outlook

Auricular cartilage reconstruction remains clinically challenging due to its complex anatomy, limited donor availability and the suboptimal long-term performance of conventional graft-based approaches. In this context, tissue engineering has emerged as a promising alternative, with bioinspired smart material systems increasingly recognized as key enablers of functional regeneration. Recent advances have driven a shift from static scaffolds toward adaptive material platforms that integrate ECM biomimicry, mechanical optimization and stimulus-responsive functionalities, enabling dynamic and spatiotemporally regulated microenvironmental modulation to support cartilage regeneration.

From an application perspective, the strategies discussed in this review show strong potential for advancing clinical auricular cartilage reconstruction, particularly in congenital microtia, post-traumatic defects and oncologic resections. Programmable control over scaffold mechanics, shape fidelity and bioactive signaling may reduce fibrosis, improve long-term morphological stability and enhance tissue integration compared with autologous grafts or inert implants. Moreover, compatibility with emerging fabrication technologies, such as 3D/4D printing and modular assembly, supports the development of patient-specific and scalable auricular constructs.

Despite this progress, several challenges must be addressed before clinical translation. Advanced scaffold designs often involve multicomponent architectures and dynamic chemistries, complicating scalable manufacturing, quality control and regulatory compliance. In addition, long-term safety, mechanical durability and immune compatibility remain insufficiently understood, particularly with respect to degradation products, phenotype stability and chronic immune responses [187]. Future efforts should therefore emphasize material simplification, long-term immunological and biomechanical evaluation and translationally oriented manufacturing strategies. The integration of modular design principles, AI-assisted optimization [188] and closed-loop regenerative systems incorporating biosensing and feedback control may further enhance the clinical reliability of bioinspired smart scaffolds.

## Supplementary Material

rbag025_Supplementary_Data
