# Supplementary material for: Bioinspired and smart material systems for auricular cartilage engineering: toward microenvironment-responsive and self-regulating scaffolds
Source: Regen Biomater. 2026 Mar 2;13:rbag025. doi: 10.1093/rb/rbag025 (PMC13070657; doi:10.1093/rb/rbag025)
Supplement: rbag025_Supplementary_Data [file rbag025_supplementary_data.zip › Supporting Information.docx]

**Bioinspired and Smart Material Systems for Auricular Cartilage Engineering: Toward Microenvironment-Responsive and Self-Regulating Scaffolds**

Yan Gong^a,^*****, Haiyue Jiang^a,^*****, Xia Liu^a,^*****


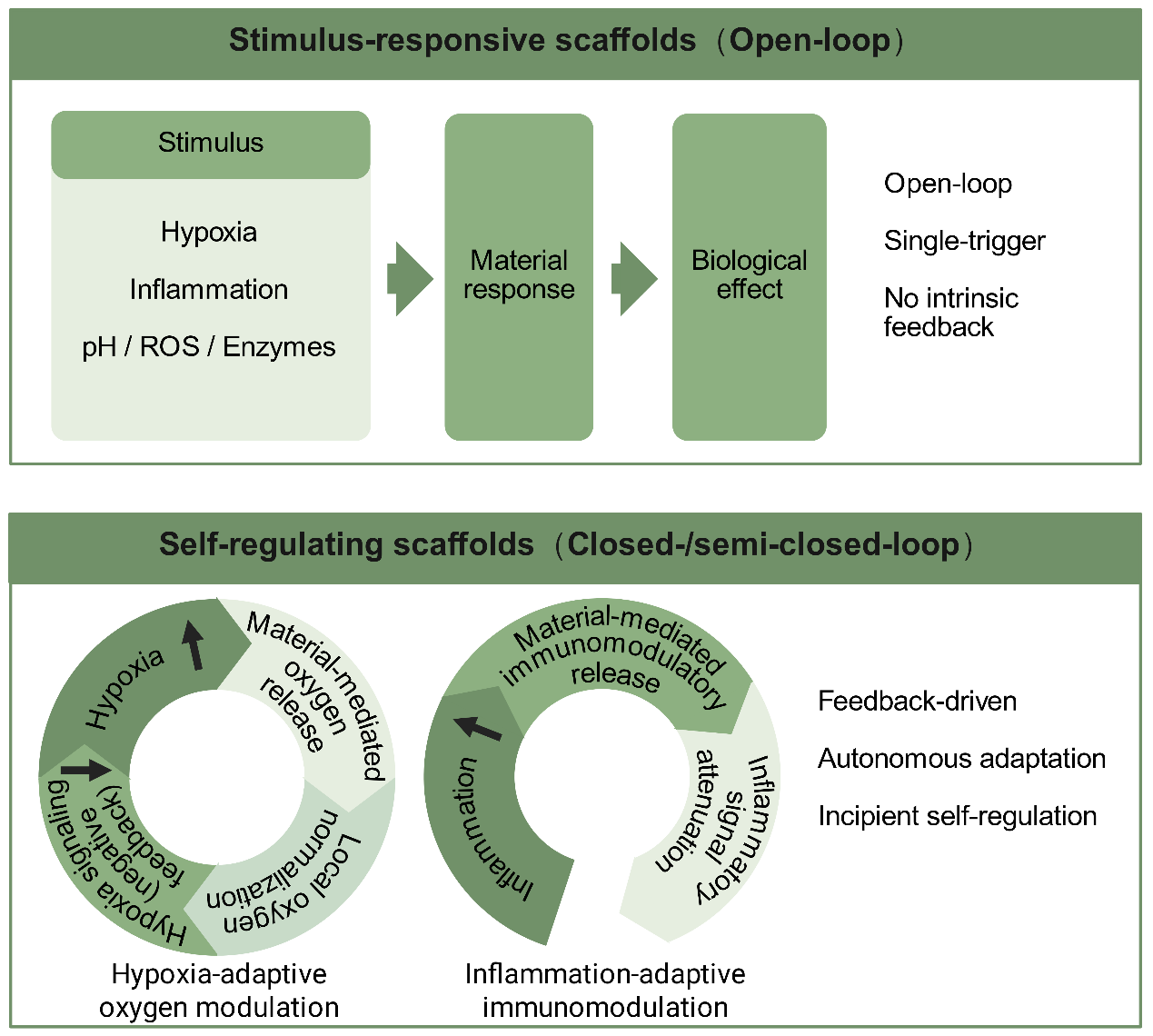


**Figure S1.** Conceptual comparison between stimulus-responsive and self-regulating scaffold systems.

Stimulus-responsive scaffolds operate in an open-loop manner, in which predefined external or internal cues (e.g., hypoxia, inflammation, pH, or enzymes) trigger material responses without feedback regulation. In contrast, self-regulating scaffolds integrate sensing, actuation, and feedback mechanisms, enabling autonomous adjustment of scaffold behavior based on downstream biological outcomes. Representative examples include bidirectional oxygen-regulating systems and inflammation-sensing immunomodulatory platforms, which establish negative-feedback loops to dynamically stabilize the regenerative microenvironment.
